# Practical guide to structuring the Discussion section in clinical and epidemiological studies

**DOI:** 10.1186/s12998-026-00645-z

**Published:** 2026-09-01

**Authors:** Charlotte Leboeuf-Yde, Claire Gudex

**Affiliations:** 1 Department of Regional Health Research, University of Southern Denmark, Odense, Denmark; 2 Research Unit OPEN, Department of Clinical Research, University of Southern Denmark, Odense, Denmark

**Keywords:** Writing process, Structure of a scientific article, Discussion section

## Abstract

This is the last of three articles that provide step-by-step guides on how to structure the content of a scientific article to ensure a coherent and relevant text. The current article aims to help authors produce relevant and stringent Discussion sections that reflect clarity of thought and critical analysis, with contents driven by the research objectives. We first describe the purpose of the six typical elements of the typical Discussion section: (i) Summary of Findings, (ii) Explanation of Findings, (iii) Comparison of own results with other studies, (iv) Critique of own work, (v) Perspectives, and (vi) Conclusion. We show different placements and combinations of these and explain what they should include. We then suggest how to write a simple Summary of Findings and how to avoid re-writing the Background section in the Explanation of Findings. We show how to use the previously identified research gaps when comparing own results with similar studies, suggest different approaches for Critique of own work, define the types of Perspectives that are common in clinical and epidemiological studies, and explain how to provide relevant and memorable information in the Conclusion. Examples of how authors have dealt with different elements of the Discussion section have been selected from this journal. Finally, we discuss the use of artificial intelligence in the writing process. We hope that the three articles will help authors to plan their writing in a meaningful manner, thus saving time for themselves and resulting in articles that are easy to read and understand.

## Introduction

This is the last of three articles on how to structure a scientific article with the aim of making it easier to produce informative and easily read reports of clinical and epidemiological studies. The first article deals with the Background section [1], and the second describes how to structure the Methods and Results sections [2].

In the present article, we first discuss the purpose of the different elements of the Discussion section and then present an approach for planning the structure and contents of the whole Discussion section as a basis for the full text. We illustrate our points with examples from articles published in this journal, showing how other authors have approached the various parts of the Discussion text in diverse ways. However, good and clear examples were more difficult to find for this section than for the previous ones!

## The purpose and content of the Discussion section

The Discussion section pulls the threads of the study together by explaining and probing the results in an honest attempt at uncovering their meaning and validity and placing them in a larger perspective. Therefore, this section is best dealt with after all the data have been processed and fully interpreted, and after you have developed a certain intellectual distance to your own work.

As in the Background section, the writing style of the Discussion section is narrative. To avoid a long and/or confusing text, the content can be anchored on the following six elements:

i) Summary of Findings.
ii) Explanation of Findings.
iii) Comparison of your results with those from other studies.
iv) Critique of your own work.
v) Perspectives.
vi) Conclusion

While the Discussion usually starts with the Summary of Findings and ends with the Conclusion, the other items can be organized in different ways. For example, the Explanation of Findings and the Comparison of results are often combined, while the Perspectives can be located in various places.

Note that the Discussion sections of published articles often have only two headings, one referring to methodological considerations (strengths and limitations) and the other indicating the conclusions. However, it would sometimes help both the writer and the reader if the other elements were also indicated in the text with suitable headings. If the journal will not accept them, we suggest you at least use them when creating the manuscript.

Here, we briefly explain the purpose and content of the six elements, after which follows more detailed practical information.

i) Summary of Findings.

This introductory paragraph is meant to remind the reader of the study findings. It should not repeat all the detailed results or include a list of exact estimates, confidence intervals, p-values, etc. unless the purpose of the study was to obtain a numerical result such as a prevalence estimate. Some authors also remind the reader of the research design and/or briefly indicate an outstanding feature, such as “*This appears to be the first RCT on this topic to use validated outcome variables”*, i.e. providing not only information on the research design but, as in this case, also emphasizing that it is the first study of its kind and that it used validated assessment tools.

ii) Explanation of Findings.

Readers are likely to benefit from the expertise you have developed during the study, so by including some of this knowledge in your Discussion, you help to make the results more easily understood and interesting. While the Background section relates to what you knew and thought *prior* to conducting the study, the Discussion section is about what you now know *after* collecting and analysing your data.This may relate to possible reasons for the asociations found (or not found), mechanisms of action, technical aspects of the study process (unless this is included under ‘Methodological considerations’), potential links, or pathways, and could include a suggestion for future use (i.e. a perspective).

iii) Comparison of your results with those from other studies.

It is tempting to write a long and detailed section here, but really what is needed is just an indication of whether your results agree with, supplement, or contradict the findings of previous studies. In other words, you should not re-write what you have already written in your Background section. You should, however, attempt to explain possible reasons for any differences between your results and those of others.

iv) Critique of own work.

The validity of your results will depend on the quality of the research process. Therefore, your reader must be made aware of any weaknesses (or absence of the same!) and their potential consequences. It is difficult to perform perfect studies, and most research projects either have design flaws or encounter problems along the way. It is best to be honest and transparent about these. Methodological weaknesses that are acknowledged by the authors are generally accepted, considered, and ‘forgiven’, whereas flaws uncovered by the reader make the authors appear untrustworthy. Note that any strengths mentioned here must be over and above what would be expected from your study design, e.g., it would not be outstanding to have used randomization in an RCT. However, you could mention that you included enough study subjects to make it possible to perform additional subgroup analyses.

v) Perspectives.

This part of the Discussion sets your study findings into the context of the wider world and indicates how they could be used or built upon. Relevant, high-quality studies typically bring something of value to (i) other researchers, (ii) health professionals and their patients, and/or (iii) the general public. All studies should have findings that are useful to the research community (e.g., suggestions for the design of future studies), but many will also have a clinical or more general public health perspective.

Do not forget that ‘negative’ results may be especially interesting as they can help direct research into more useful areas, stop irrelevant clinical procedures, or change useless policies—in which case they should be mentioned in the Perspectives. Note that if you are reporting a validation study or pilot study as a preliminary step towards a main study, the perspective here may relate only to your own needs for the main study.

vi) Conclusion.

The Discussion section ends with the Conclusions, which refer to the results for each research objective but with few (if any) statistical details, thus attempting to take the big picture into consideration. The conclusion(s) must be informative and preferably brief, making it also suitable for repeating in the Abstract. Remember that many people read only the Title and the Conclusions of an article, so make these easily understandable and memorable.

## Ordering the text of your Discussion section

When planning the structure of your Discussion section, you should first look at your research objectives. These will guide you on what to write about throughout the whole Discussion section, from the Summary of Findings through to the Conclusion.

As indicated above, the Discussion section can be organized in different ways where the four ‘middle’ items can be placed in various orders between Summary of Findings and Conclusion:

- Explanations of Findings and Comparisons to other studies may be separate paragraphs or combined, and they typically come just after the Summary of Findings.
- Critique of own work typically comes after Comparison to other studies; however, if there are important concerns about the quality of the study, the reader should be made aware of these major limitations before reading more about the study results—in this case, the Critique of own work can be given a prominent place ímmediately after the Summary of Findings.
- The Perspectives can be placed in various locations: (i) Often, they are assembled under one heading “Perspectives” and placed close to the Conclusion; (ii) They may also be combined with the Conclusion without a specific heading; (iii) Different types of perspectives can be split up, e.g., clinical or public health perspectives could be added to the Explanation of Findings, while research perspectives could be added to the Critique of own work.
- If different topics need to be discussed separately because they use different arguments and references, you can structure the Discussion in ‘slices’. After a general Summary of Findings at the start of the Discussion section, each topic slice could then include its own Summary of Findings, Explanations of Findings, Comparison with other work, and perhaps Perspectives also. The Conclusion (for the whole article) would finish the section.

If your Discussion section grows out of proportion, it is probably because you have strayed from your research objectives, repeated some of the information you provided in the Background section, or included too much information on other studies when comparing their findings to yours. To avoid this, we recommend that you follow the structuring guide below.

## A practical guide to structuring your Discussion section

### Use your research objectives to plan the structure

Review your research objectives to remind yourself of what sort of information you need to write about. Then list the six elements of a typical Discussion section as your main headings and decide in which order you want to present them:

- Summary of Findings goes first and Conclusion last.
- Decide if you will combine Explanation of Findings and Comparison with other studies or write them separately.
- Determine if your methodological limitations are so important that you should mention them before explaining the detailed findings.
- Consider your Perspectives and how to place them.
- If your study deals with several separate subjects, you may wish to include several of these headings under various ‘slices’; if so, decide which information goes under each ‘slice’ and which information will be discussed under the general topics.

This initial structure may look complicated and requires some reflection, but it will help you to develop a relevant text with a good flow. You can remove some of the headings later when you have written the text.

## Summary of findings

For this first element, write a summary of the results for your research objectives, simply providing an ‘answer’ to *each* of your research objectives or questions. Use the same words for key aspects such as the study context/population and your variables of interest. This section is best done by summarizing the results for each research objective in simple words, not forgetting any disappointing or negative results. Be careful not to mention only the positive (or negative) findings to the exclusion of the other.

We suggest you write this section in complete sentences, even at this stage, to ensure that you cover all the findings from your study and that you do not include too many statistical details. Note that the Summary of Findings only reports the results—it does not explain them.

It is usually easiest for the reader if the order of results in the Summary of Findings is the same as the order of research objectives in the Introduction section. If you find that your analysis has deviated from your original research objectives, you need to either add a research objective or remove an analysis! Examples of the Summary of Findings in published articles are provided in Table 1, together with an indication of how the the Summary of Findings can be linked to the Research Objectives.

**Table 1:** Examples of Summary of Findings (SoF) in the Discussion sections of published articles, with our own analysis indicating links with the research objectives (ROs) (numbering added if not in original text) and any additional text (underlined)

Research objectives (taken from the Introduction section)	Summary of Findings text The underlining and numbering of statements were added by the present authors	Analysis of structure
“… Specifically, we aimed to investigate (1) whether patients change their analgesic use from before to after the intervention, (2) if self-efficacy at baseline affects analgesic use at follow-up, and (3) whether improvement in self-efficacy from before to after the intervention modifies the effect of baseline analgesic use on future analgesic use” [3].	“Our results provide insights about the role of preexisting analgesic use and self-efficacy for continued use of analgesics among patients with persistent LBP in primary care. (1) Most participants did not change their analgesic use from before to after the standardized patient education and exercise therapy programme, however, 35% did discontinue analgesic use. (2) People who used analgesics when entering the care program had substantially higher odds of using analgesics after the intervention, while those with higher self-efficacy at baseline had lower odds. (3) Although we did not find a positive change in self-efficacy to modify the association between analgesic use at baseline and follow-up on the multiplicative (log-odds) scale, we found it to attenuate the absolute risk of continued analgesic use among analgesic users at baseline (i.e., negative additive interaction).	In the Introduction section, the three ROs are clearly separated by numbers 1–3. The Discussion section starts with a reminder of the study topic (underlined) and then the SoF summarizes the results of the three ROs in the same order, making it easy to link these two aspects of the text.
“…Yet, little information is available regarding the French chiropractic workforce, including their (1) sociodemographic characteristics, (2) professional activities, (3) work organization, (4) clinical practice patterns, and (5) interprofessional referrals. This study aims to address this knowledge gap by providing the first comprehensive job analysis of the chiropractic profession in France” [4].	“This study provides the first comprehensive description of French chiropractors’ profiles and activities, patients seeking chiropractic care, and patterns of interprofessional referrals. (3) Just over a quarter of participating chiropractors worked in a multidisciplinary setting. They reported (5) referring patients to other healthcare professionals, particularly general practitioners, more frequently than they received referrals from them. (4) Middle-aged adults were the most common patients seeking care, and spinal pain was the main chief complaint. (4) Treatment modalities generally consisted of manual therapy not restricted to HVLA manipulations, advice, patient education, and therapeutic exercises.”	In the Introduction section, the RO is preceded by a list of specific research gaps, each of which could represent a RO. The Discussion section starts with a reminder of the study aim (underlined) and then the SoF presents the major findings (without unnecessary details) for 3 of the 5 gaps, and in a different order than earlier.
“This trial’s primary objective was to determine the effects of MTrP-DN compared to S-DN in a MRh on postoperative (1) shoulder pain for patients following RCR. The secondary objectives were to determine the effects of MTrP-DN compared to S-DN in the MRh in both groups on (2) ROM, (3) shoulder muscles’ strength, and (4) SPADI. We hypothesized that the patients who received MTrP-DN as part of their MRh would exhibit greater improvements than those who received S-DN instead.” [5].	“According to the findings of our study, implementing 4 sessions of MTrP-DN for shoulder girdle muscles in the MRh did not show statistically greater improvement in (1) shoulder pain, (2) ROM, (3) strength, and (4) SPADI than adding a sham intervention.”	The SoF follows the same order as in the ROs provided in the Introduction section and covers all four ROs.
“Therefore, the aim of this study was to investigate associations between (1) demographic, (2) anthropometric, and (3) SMT experience factors and (A) confidence and (B) competence in delivering specific SMT force–time characteristics on mannikins.” [6].	Our study aimed to investigate the associations between demographic, anthropometric, and SMT experience factors and chiropractic students’ confidence and competence in delivering specific SMT force–time characteristics. We found that (A) confidence varied significantly with (1) males and (3) students in later years in the chiropractic program showing higher confidence levels. Likewise, (1) (3) these groups also had higher odds of (B) performing SMT with time to peak force below 150 ms, and achieve [sic] the 800 N peak thrust force. However, no other force–time characteristic showed a strong relationship with sex or year of study. Additionally, (2) taller students, regardless of sex, had higher odds of (B) achieving the 800 N peak thrust force, a target force that demonstrated the highest variability and posed the most challenge for students. Furthermore, while time spent in the FSTT^®^ lab significantly influenced (A) confidence, it did not translate into higher (B) competence.”	The Discussion section starts with a reminder of the study aim (underlined) and then the SoF reports the results for each RO listed in the Introduction section, albeit in a slightly different order to present similar results together.

## Conclusion

It is useful to write the Conclusion immediately after the Summary of Findings to avoid the two becoming (near) identical. The Conclusion usually considers a wider picture than the Summary of Findings and is often related to the overall goal of the study rather than the specific and detailed objectives. If it is difficult to write an informative Conclusion, it might be easier to refer back to the overall study aim and main finding (and perhaps the study design) and then add text on Perspectives. Nevertheless, if your study is very simple, the Conclusion may end up very similar to the Summary of Findings. Because many people skip to the Conclusions of the Discussion section, we recommend that you avoid abbreviations here. Different ways of presenting the Conclusions are shown in Table 2.

**Table 2:** Examples from published literature of the Conclusions text, indicating (by underlining) whether it includes a reminder of the study aim, the study design, or a Perspective. All examples were located under the heading “Conclusion” at the end of the Discussion section

Conclusion text	Comments
“This study found that while older chiropractic patients with LBP had slightly higher disability scores and a higher prevalence of non-MSK comorbidities, age was not the strongest factor associated with disability outcomes. Instead, baseline disability, depression, self-perceived general health, and MSK comorbidities were more consistently linked to higher disability scores across all follow-up time points. These findings suggest that age should not be viewed as an isolated risk factor for prolonged disability, but rather as one part of a broader bio-psychosocial framework” [7].	This Conclusion text finishes with a clinical Perspective.
“The results of this study show that both IMES and DN treatments can have positive and promising effects of improving UT TrP. Intramuscular electrical stimulation is more effective for some clinical symptoms and ultrasound parameters compared to DN with more permanent effects. To examine the effects of these interventions in more detail and to compare them with other common treatments and to examine the effects of this intervention on people’s lifestyles and performance, more studies with larger sample sizes are necessary.” [8].	This Conclusion text finishes with a research Perspective.
“Using AI-generated case vignettes, this study found that contextual factors of aging had varying impacts on chiropractors’ kinetic and kinematic characteristics when delivering SMT. This suggests that clinicians differentiate between chronological, pathological, and felt age, and use that information to inform the kinetic and kinematic parameters of FBM administered to older adults. Future research is needed to identify ideal kinetic and kinematic characteristics to achieve desired clinical outcomes based on various aspects of aging, and train providers of SMT to utilize contextual factors to individualize care.” [9].	This Conclusion starts with the study design (underlined), continues with Conclusions and ends with a research Perspective (underlined).
“In sum, the aims of the present study were to explore patients’ perceptions and preferences regarding the attire worn by chiropractors in a university clinic. Overall, results suggest that scrubs are the most preferred attire, while casual attire is the least preferred. Chiropractors wearing scrubs received the highest scores of perceived knowledgeability, trustworthiness, competence, professionalism, and ability to make patients feel comfortable. The formal attire and the white coat also received high scores on these perceived competence and skills domains. More research is nonetheless needed to understand how a number of moderators, such as providers’ and patients’ age, lifetime exposure to chiropractic treatments, and clinic settings, influence patients’ perceptions and preferences.” [10].	This Conclusion text starts with the study aim (underlined) and ends with a research Perspective (underlined).
“The Danish Headache Questionnaire has good content validity and is a feasible tool for measuring clinician characteristics and knowledge when managing patients with headaches and obtaining prevalence estimates in Danish chiropractic care and has the potential to be a useful tool for primary care in general. Furthermore, it can be used as an educational tool useful to guide a complete and standardised assessment. The generic version could be used in diverse primary care settings, ensuring a more standardised approach and facilitating comparisons between settings. These findings suggest that the Danish Headache Questionnaire may be a valuable instrument for clinical assessment in primary care and have the potential to fill in gaps of knowledge which could improve the management of headache disorders in primary care. Furthermore, future studies could use the knowledge gained in randomised controlled trials on the effectiveness of management and treatment in primary care. This could give a deeper understanding of the most beneficial management and treatment strategy for individuals suffering from headaches” [11].	This Conclusion text starts with the main study result followed by the underlined text that consists of clinical Perspectives (use in primary care, educational tool) and research Perspective (randomized controlled trial).

## Explanation of Findings

It is important to avoid a long and confusing explanation of findings. Only provide relevant information in relation to your results by including only items that help the understanding of your results, i.e., aspects that may not be obvious to the reader. Importantly, ensure that you do not repeat your Background section by mentioning the same facts.

## Comparison of own results with those from other studies

This part of the Discussion section is often long and confusing in published articles. An elegant strategy to avoid this is to make use of the *research gap(s)* that you identified when planning the study. These were described in your Background section [1] and have the following consequences for the Discussion (some examples from the literature are provided in Table 3):

**Table 3:** Examples from the literature of different approaches to comparisons with previous studies, in relation to the type of research gap in the Introduction section

Type of research gap	Text in the Discussion section	Comments
Some knowledge in this area, but some evidence is missing (‘jigsaw puzzle’)	*“….this finding aligns with the results of Hossler and colleagues [22]… “As suggested by Hossler and colleagues [22] …”* [10]	Results in line with previous studies
	*“Our credibility and expectancy findings are in line with those of a recent RCT of SMT for back pain [40].”* [12]	Results in line with previous studies
Contradictory findings—current results agree with one side but not the other (‘ping-pong’)	*“This is consistent with a study by Fu et al. [35]…”* *“We found that …*,* which [is] contrary to other literature that reported that …[7*,*8*,*36].”* [7]	Some results are in line with previous studies, other results are not
	*“This difference in gender was consistent with previous studies involving trainees [30*,*31]*,* but contrasts with another study involving experienced clinicians [32]”.* [9]	Some results are in line with previous studies, other results are not
No previous research (a ‘hole’)	*“To the best of our knowledge*,* this study is the first one to compare the effectiveness of IMES and DN interventions on patients with active TrP in the UT muscle using clinical and sonographic parameters.” (placed in the Summary of Findings)* [8]	No previous similar study, so comparison studies use other result variables
	*“…to our knowledge*,* there exists no validated questionnaire specifically designed to assess the management of patients with headaches in primary care”* [11]	No previous similar study, so nothing to compare with

- A ‘*jigsaw puzzle***’** situation requires an explanation of how your new results fit in with the pre-study jigsaw picture. This means that you refer only to studies that are relevant, and you do not need to discuss the ‘outside pieces’ of the jigsaw puzzle. “*These findings fit well with….*” or*“These results help provide a more complete picture…”*.
- When previous studies have reported contradictory findings (‘*ping-pong*’), you can simply state that your results agree with specific studies (listing the references without going into further detail about the studies). Thereafter, you list the studies that your results did not agree with and attempt to explain why this might be. Typical reasons for disagreement between studies are differences in study design, study population, sample size, definitions and cut-off points, and statistical approach and varying quality of research.
- If there was a **‘***hole***’**, there is obviously nothing to compare with. *“As we could not find any similar studies*,* we cannot compare our results to those of other studies.” or “To our knowledge*,* no similar studies have been published…”*.
- If you worked with a *clinical myth*, there might not be any scientific work to refer to, but perhaps you have found some descriptions of what people believe or do. Otherwise, you can hold up your research findings against what is “commonly known”, “commonly seen in clinical practice”, “commonly taught”, or whatever it was that triggered your curiosity about the topic.
- There are only a few other reasons for doing a study, one being that *new technologies* were available. You would then have to compare your new findings with the old ones. Another reason could be that you needed to do some *preliminary validation* before a main study (e.g., inter-examiner reliability). In this case, you will find that your Conclusion will be similar or identical to the Summary of findings as there is little more to say.

Note that it is in the Discussion where you can refer to studies that emerged *after* you planned your own study (i.e. you should not write about new information in the Background section). Thus, if an identical study to yours emerged after you started your own, there is no need to panic. The reasons for conducting your study are intact, and you use the new study to compare their result to yours. If they agree, these studies together strengthen this evidence and if they disagree, you can include an insightful discussion on potential reasons for this. “*During the course of our study*,* similar work was published agreeing/not agreeing with some of our findings…”*.

## Critique of own work

In this part of the Discussion section, you typically list the (potential) weaknesses of the study methods, explain their likely consequences, and perhaps suggest any (future) remedial actions. However, this is also the place where you discuss any outstanding methodological aspects of your work, or explain what you did to mitigate any inherent weaknesses in your study design. The term you choose for the critical review of your own work is determined by how you approach it.

- ‘Methodological considerations’ is a broad term that allows you to discuss both positive and negative aspects of your study methods and to deal with practical issues, e.g., why you chose one approach over another, how a specific difficulty was overcome (or not), how you developed a new test, or how a procedure should be conducted or interpreted within the study.
- The term ‘Strengths and limitations’ allows you to discuss both positive and negative aspects of your study methods.
- ‘Study limitations’ obviously allows you to discuss only the negative aspects of your study methods.

Irrespective of which term you choose, you need a clever approach to select which items to discuss. We suggest the following:

- Go back to the plan for your Methods section. At each heading, stop and think about any potential data quality or bias issues that an insightful reader might detect. List these issues and make short notes for each in turn. 
- For each issue, consider whether it is best to explain your approach in the Methods section rather than dealing with it in the Discussion section. If there is a real weakness or limitation in your study methods, do not try to hide it but help the reader by identifying any weaknesses. Honesty always looks better in research than attempts at concealment.
- Classify your items into (i) a real weakness with consequences, in which case you must explain what these consequences might be, (ii) a real weakness but with unknown consequences, then say so, or (iii) a minor or irrelevant weakness with no obvious consequences, which you should also explain.
- If you knew in advance of a weak point in your study, you might have undertaken some extra activities, tests, or analyses to negate or lessen any negative effects. If so, you can also explain these here.

Table 4 provides some examples from the literature of different approaches to Critique of own work in the Discussion section. The methodological strengths and weaknesses listed in the published articles are provided to illustrate the types of items that can be relevant. Most authors point out the possible consequences of limitations, and some provide attenuating arguments against the limitation being a serious flaw. One of these examples includes research Perspectives within the list of limitations.

**Table 4:** Examples from the literature of different approaches to Critique of own work in the Discussion section

Heading used in the text	Number and type of strengths and limitations described
“Methodological considerations” [6]	Four limitations are explained, and the possible consequences of each are discussed (shown in parentheses): (i) Cross-sectional design (limiting ability to identify prognostic risk factors), (ii) Participant recruitment through convenience sampling (selection bias), (iii) Unmeasured confounding variables (with examples), (iv) Single-centre study (may limit generalisability of results).
“Strengths and limitations” [4]	One strength noted: a large proportion of listed chiropractors were invited to participate and achieved a reasonably high response rate. Four limitations are explained with possible consequences, and two with attenuating arguments: (i) The survey was long, which likely contributed to participant dropouts, (ii) Recruitment strategy may have introduced selection bias–“Still, the consistency between several of our findings and those of similar job analyses…suggests that the present survey provides a realistic portrait of the French chiropractic profession….”, (iii) Self-reported data may be prone to recall and social desirability biases–“However, the results obtained for such items suggest that participants provided honest responses”, (iv) Some data collected as ranges rather than precise values, allowing only for rough estimates.
“Weaknesses” “Strengths” “External validity” [13]	Four weaknesses are explained with possible consequences, and one with attenuating arguments: (i) Clinicians performing the interventions could not be blinded to the group allocation (could have favoured the intervention group)–“but the risk was considered low, considering that the therapists received written and verbal instructions on how to communicate and treat the study participants.” (ii) Most participants had previously experienced a positive effect from chiropractic treatment (possible selection bias), (iii) Did not record whether the patients continued the stretching exercises in the individualized care period, (iv) Recruitment occurred 5 weeks before study start and many had less neck pain at study baseline (potential floor effect). Four strengths listed, one with possible consequences: (i) Well-controlled RCT, (ii) High response rate, (iii) Blinding of the participants to others’ interventions, and of the research assistant and statistician to the group allocation, (iv) Gave both groups the same time and attention (assuring minimal differences in contextual factors). External validity described as good because “The study group included individuals with all levels of NP to encompass the diverse group of persistent or recurrent NP patients.”
No heading but in the text: “Our study has strengths” and “Our study also has limitations” [12]	Seven strengths are briefly listed, mostly with consequences: (i) Prespecified standard operating procedures to maximize intervention fidelity and balance contextual effects, (ii) Blinding to foster impartiality, (iii) Manual placebo intervention as comparator, (iv) Real-world setting with high patient retention, v + vi) Further details about blinding, (vii) Qualitative thematic analysis to explore possible influencing factors. Four limitations are listed, two with attenuating arguments and one with possible consequences: (i) Deviation from protocol, (ii) Three participants did not receive the allocated intervention–did sensitivity analyses to explore the impact, (iii) imbalance of participant gender at baseline–did post hoc blinded analysis of gender differences, (iv) study population has mild intensity LBP at baseline (may limit generalisability of results).
“Limitations” [5]	Seven limitations are listed, of which five have a research Perspective inserted: (i) The needling technique in the control group may have reduced pain levels–“…highly recommend the use of non-penetrating blinding strategies in the control group for future controlled trials”, (ii) Short follow-up of 4 weeks– “…recommend long-term follow-ups with more sessions of MTrP-DN for postoperative shoulder pain”, (iii) Inability to control concomitant treatments, iv) Single-centre study (possibility of contamination bias)–“…suggest the feasibility of multi-center investigations on MTrPDN for the patients following RCR surgery”, v) Some potential confounders not considered–“ …recommend considering these factors in future investigations”, (vi) Sample with wide age range– “… recommend future studies implement subgroup analysis for age or design trials with narrower age ranges”, (vii) The trial is preliminary and small sample size.

## Perspectives

For this section, you need to consider the implications that your study results might have for the outside world. Think about how your study findings contribute in terms of these three main types of perspectives:

- *Research perspectives*: Here you can indicate useful directions for further research so as to help others avoid your mistakes and not waste time and resources. However, do not simply write “More research is needed”. Such a statement is unhelpfully vague and needs to be supplemented by specific recommendations, e.g., how to avoid some of the shortcomings of your own study or how to test the generalizability of your study findings. You could, for example, suggest amendments to the sample size or selection criteria, the type of data collected, or the analytical approach.
- *Clinical perspectives*: Results that are of interest to health professionals (and perhaps also the health service users) should be highlighted here. This relates to any knowledge that could affect the behaviour or attitudes of health professionals and patients, e.g., recognition of patient characteristics, choice of diagnostic or management approach, perceptions of health and illness, interpersonal encounters, decision-making.
- *Public health perspectives*: These are your research findings that could be of interest for the population at large by changing concepts or procedures on a larger scale, e.g., vaccination programmes, health policies, resource allocation, exercise and dietary guidelines for the general population.

Different approaches to the writing of Perspectives are shown in Table 2, all under the heading ‘Conclusion’.

## Move your Conclusion

Your Conclusion can now be moved down to its rightful place at the end of the Discussion section. Decide whether it will stand alone or if it will have one or more Perspectives added to it.

## Review the structure and content of your Discussion section

Finally, check the structure again and some of the content of your Discussion section using the tips in Text box 1.

Text box 1. Tips for checking the content once you have structured your Discussion section

- In your Summary of Findings, have you referred to all your research objectives (even negative findings)?
- Have you covered the Explanations of Findings and Comparison to other studies without writing too much?
- Have you given an appropriate heading to the ‘Critique of own work’ paragraph, and have you considered the potential consequences of your study limitations?
- Have you indicated at least one perspective for your study results (research, clinical, or public health)?
- Check your references: is it easy for your reader to see which statement each reference belongs to? Have you included only relevant references, i.e. those that reflect the information you are providing (usually the most recent or best quality references)?
- Can your Conclusions be used as an informative part of the Abstract?

## Using AI for the Discussion section

Most researchers have probably realized that artificial intelligence (AI) can be used for more than just language editing. Generative AI is now widely available and increasingly used as a writing aid. It can be a valuable tool for starting the writing process, for getting ideas on how to approach the task, and to check the grammar and flow of your own writing. Grammarly, for example, can identify grammatical errors and improve style, but note that it can be challenged by complex technical and scientifc terms, meaning that authors need to carefully check any changes made [14].

AI can be useful in interpreting and summarizing text, but it can also make considerable errors, including citing sources that do not exist or fabricating information (‘AI hallucination’) [14, 15]. Therefore, generative AI must be used alongside human intelligence, especially as the human author is responsible for the contents of an article, not the AI model. As an author, you must decide whether you want to ignore the use of generative AI or to use it for specific tasks. If you do use it, make sure you apply your own critical oversight to ensure rational explanations and to improve your own intellectual capacities. Create your own structure and content first, thinking about the specific needs of your study, before asking AI for feedback. This will help you to retain ownership of your manuscript and to develop your critical thinking skills.

When writing up your study, it is important to describe how you have used AI—whether it was at the planning stage, during data collection or analysis, or while preparing your manuscript. Advice on how to report the use of AI in scientific articles was given in the articles on the Background [1] and Methods/Results [2]. In September 2025, the International Association of Science, Technical & Medical Publishers (STM) published a classification framework for how authors might use generative AI in academic writing [16]. This will be a further aid to authors in reporting AI use.

## Resources to help you learn new skills

Rather than going directly to generative AI to help you draft and correct your text, consider using the two resources below. They are tools that will support your further learning so that you will find it easier to write your next article.

*The Medical Writing Checklist* (www.sdu.dk/en/WRITE).

This simple English grammar checklist helps you go through your manuscript item by item, checking for central aspects of English grammar. Topics include checking sentence length, removing unnecessary words, handling of abbreviations and apostrophes, and use of commas. Clicking on a topic takes you to more a more detailed explanation with text examples from the health sciences.

The checklist is available as a free phone app (search for ‘Medical Writing Checklist’ with the green tick mark) or can be downloaded from the WRITE website of the University of Southern Denmark.

*The Academic Phrasebank* (https://www.phrasebank.manchester.ac.uk/).

This website is a fascinating source of elegant scientific English phrases, categorized by the sections of a scientific article (Introduction, Methods, Results, Discussion, Conclusions) or by the text’s function (e.g., being critical, comparing and contrasting, explaining causality). The examples provided range from shorter to longer phrases. For most scientific texts, the shorter and less wordy options are preferable.

## Summary

The purposes of the Discussion section are to present and interpret the study findings in an easy-to-read format for your readers, to present any methodological issues in a transparent and honest manner, to indicate the potential usefulness of the study results, and to point researchers into relevant directions. This often makes it long and heavy with information, but it can still be interesting to read if you separate it into meaningful parts. A clear and helpful Discussion section requires planning and depends on well-formulated research objectives. We hope that the guideline provided here for structuring your Discussion section will help you produce a complete but relevant text that reflects all the hard work that preceded it. Remember that the Discussion section is where you show your critical analysis skills—whether you use AI or not, the intellectual property and responsibility of the article is yours.

This is the last of our three articles on structuring the content of data-driven scientific articles for reporting clinical and epidemiological studies. We hope you find them useful for answering the questions that many of us have when planning a scientific manuscript: What content is relevant for this paper? Where should I place the various pieces of information? How do I develop a red thread to make a cohesive story?

Our very last tip is to remind you to change your ‘hat’ when deciding on the structure and content of your report. Rather than focusing on your own needs and interests, think of the needs of the reader: What do they need to know to be able to understand your study, and how can you make it easy for them to find the most important pieces of information?

## Data Availability

No datasets were generated or analysed during the current study.

## Declarations

### Use of artificial intelligence tools

AI (Word Editor) was used to suggest spelling and grammar improvements in the text.
